# Effect of a low-intensity, self-management lifestyle intervention on knee pain in community-based young to middle-aged rural women: a cluster randomised controlled trial

**DOI:** 10.1186/s13075-018-1572-5

**Published:** 2018-04-17

**Authors:** Yuanyuan Wang, Catherine Lombard, Sultana Monira Hussain, Cheryce Harrison, Samantha Kozica, Sharmayne R. E. Brady, Helena Teede, Flavia M. Cicuttini

**Affiliations:** 1 Department of Epidemiology and Preventive Medicine, School of Public Health and Preventive Medicine, Monash University, 553 St Kilda Road, Melbourne, VIC 3004 Australia; 2 Monash Centre for Health Research and Implementation, Monash University, Melbourne, Australia; 3 Department of Nutrition and Dietetics, Monash University, Melbourne, Australia; 4 Diabetes and Vascular Medicine Unit, Monash Health, Melbourne, Australia

**Keywords:** Randomised controlled trial, Lifestyle program, Knee pain, Rural women, Weight

## Abstract

**Background:**

Knee pain is common with obesity and weight gain being important risk factors. Previous clinical trials have focused on overweight or obese adults with knee pain and osteoarthritis and demonstrated modest effects of intense weight loss programs on reducing knee pain despite very significant weight loss. There has been no lifestyle intervention that targets community-based adults to test its effect on prevention of knee pain. We aimed to determine the effect of a simple low-intensity self-management lifestyle intervention (HeLP-her), proven in randomised controlled trials to improve lifestyle and prevent weight gain, on knee pain in community-based young to middle-aged rural women.

**Methods:**

A 1-year pragmatic, cluster randomised controlled trial was conducted in 649 community-based women (aged 18–50 years) to receive either the HeLP-her program (consisting of one group session, monthly SMS text messages, one phone coaching session, and a program manual) or one general women’s health education session. Secondary analyses were performed in 390 women who had knee pain measured using the Western Ontario and McMaster Universities Osteoarthritis Index (WOMAC) at baseline and 12-month follow-up. “Any knee pain” was defined as a WOMAC pain score ≥ 1. Knee pain worsening was defined as an increase in WOMAC pain score over 12 months.

**Results:**

Thirty-five percent of women had “any knee pain” at baseline. The risk of knee pain worsening did not differ between the intervention and control groups over 12 months. For women with any knee pain at baseline, those in the intervention arm had a lower risk of knee pain worsening compared with those in the control arm (OR 0.37, 95% CI 0.14–1.01, *p* = 0.05), with a stronger effect observed in women with body mass index ≥ 25 kg/m^2^ (OR 0.28, 95% CI 0.09–0.87, *p* = 0.03).

**Conclusions:**

In community-based young to middle-aged women, a simple low-intensity lifestyle program reduced the risk of knee pain worsening in those with any knee pain at baseline, particularly in those overweight or obese. Pragmatic lifestyle programs such as HeLP-her may represent a feasible lifestyle intervention to reduce the burden of knee pain in the community.

**Trial registration:**

ACTRN12612000115831, registered 24 January 2012.

**Electronic supplementary material:**

The online version of this article (10.1186/s13075-018-1572-5) contains supplementary material, which is available to authorized users.

## Background

Knee pain is a common problem which can be experienced by people of all ages, leading to physical disability and impaired quality of life [1, 2]. In the general population approximately one in five people report knee pain lasting at least 1 day during the past month [3]. In primary care, the knee is the second most common individual region of musculoskeletal pain, after the back, accounting for 10% of all musculoskeletal consultations [4]. Knee pain is more prevalent and severe in women than men [2].

The causes of knee pain are complex and multifactorial, with strong evidence suggesting that obesity is an important risk factor with a large population-attributable risk [3, 5]. We have shown in community-based adults that moderate weight gain (5%) was associated with development and worsening of knee pain [6]. Adults tend to gain weight progressively through middle age, with the modest accumulation of weight over time increasing obesity in the community [7]. For example, the average weight gain in the US population has been reported to be 0.45–0.9 kg [8] or 0.5–1.0 kg [7] per year based on data from different studies. In Australia, young and middle-aged women gain an average of 0.57 kg and 0.5 kg weight per year, respectively [9]. Given the steady weight gain that is occurring in many countries and the role of weight gain in the development and worsening of knee pain, community interventions aimed at weight loss or preventing weight gain may have a role in reducing the prevalence and burden of knee pain. Moreover, early intervention for the prevention and treatment of knee pain is particularly important, as an episode of knee pain predicts future recurrence [10].

Obesity is a well-established major modifiable risk factor for knee osteoarthritis [11]. The available evidence for weight loss is based on previous randomized controlled trials examining the effect of weight loss programs on knee pain and structural progression in populations of obese adults with established symptomatic knee osteoarthritis. Despite achieving substantial weight loss of 10% or approximately 10 kg, these trials have demonstrated modest effects on knee pain [12, 13] and no effect on structural progression of osteoarthritis [14]. For example, less than 40% of individuals reported no or little knee pain at the end of the trial, and only 14% of participants had a 50% reduction of their knee pain [12]. By the time an individual has symptomatic knee osteoarthritis, they are late in the trajectory from health through to end-stage joint disease where costly knee replacements are the only treatment option. Furthermore, these programs involve very intensive and complex interventions delivered by a multidisciplinary team of professionals to achieve significant weight loss, and therefore affordability and adherence are affected and long-term outcomes are often poor [12, 15]. There is an urgent and unmet need for a new approach aimed at preventing knee pain at a very early stage since there is evidence that any knee pain is a predictor of further episodes of knee pain [10]. Such a disease cycle is consistent with the causes of knee pain being multifactorial [3, 5] so that knee pain per se is also associated with weight gain and quadriceps muscle wasting [10, 16], with each also a risk factor for knee pain [6, 17]. This disease paradigm is also on the pathway to developing knee osteoarthritis [11].

Given the role of weight gain in the development and worsening of knee pain [6], a low-intensity intervention aiming at preventing weight gain has the potential to be of benefit in reducing knee pain. There is some evidence that a healthy lifestyle (healthy diet and exercise) may help prevent excess weight gain and maintain body weight at healthy levels, potentially providing a feasible approach to preventing and treating early knee pain before it becomes a major clinical problem [18]. However, there has been no lifestyle intervention that targets community-based adults to test its effect on prevention of knee pain. There is an urgent and unmet need for a simple low-intensity intervention for preventing weight gain and reducing knee pain along with other comorbidities, to be applied in community settings, with the aim of reducing long-time pain and disability.

A low-intensity, self-management lifestyle intervention, the Healthy Lifestyle Program for women (HeLP-her), was designed to prevent weight gain based on the self-determination and social cognitive behavioural theory, with motivational interviewing the primary method of interaction with participants [19, 20]. The HeLP-her has been tested in two prior randomised controlled trials in reproductive aged women both showing efficacy in prevention of weight gain compared with controls who received general health information only [20, 21]. Our pragmatic, cluster randomised controlled trial showed that HeLP-her again prevented weight gain and improved diet quality and self-management behaviours over 1 year in community-based young to middle-aged women in rural settings (HeLP-her rural) [19]. The mean weight change was − 0.48 kg in the intervention group and + 0.44 kg in the control group, with a between group difference − 0.92 kg [19]. The intervention group reported increased self-management strategy use related to diet and physical activity [19]. Therefore, the aim of the current knee pain substudy was to examine whether HeLP-her rural had an effect on reducing knee pain in community-based young to middle-aged women. The hypothesis was that the HeLP-her program would prevent knee pain in community-based women who were selected without reference to knee symptoms, via its effect on preventing weight gain. We tested this hypothesis by examining the effect of a sample, low-intensity intervention, which has shown an effect on weight gain prevention [19], on prevention of knee pain in community-based rural women. We selected rural women as they have a high prevalence of knee pain and reduced access to healthcare resources.

## Methods

### Study design, participants, and randomisation

A 1-year pragmatic, cluster randomised controlled trial was performed in 41 Australian towns (clusters) which were randomised using a computer-generated randomisation list for intervention (*n* = 21) or control towns (*n* = 20) [19]. Participants were recruited as clusters according to the town of residence between September 2012 and April 2013 [19]. Eligibility criteria included females, age 18–50 years, and residing in or near participating towns. Exclusion criteria were minimal and included pregnancy or serious medical conditions that would inhibit full participation in the program [19]. There was no reference to musculoskeletal conditions, including knee pain, in the inclusion or exclusion criteria. Further details were described in the trial protocol [22] and the Consolidated Standards of Reporting Trials (CONSORT) flow diagram [19]. Due to the nature of the trial, researchers were aware of group allocation at baseline. Participants were not aware of group assignment, although they were aware that they were participating in a healthy lifestyle research program. At the 1-year data collection point, both participants and new field researchers were blinded to group allocation and previous anthropometric measures, with statistical analysis completed by a blinded biostatistician [19]. The study was approved by the Monash Health Human Research Ethics Committee (Project No. 12034B). All participants gave written informed consent. The trial was registered with the Australian New Zealand Clinical Trials Registry (ACTRN12612000115831, registered 24 January 2012) prior to recruitment.

### Intervention

The details of the 1-year self-management lifestyle intervention (HeLP-her) have been published previously [19, 22]. Its key features included community integration, nonprescriptive simple health messages, small changes to behaviour, low participant burden, goal setting, self-monitoring including self-weighing, and delivery including a mix of a single face-to-face group session, one session of phone coaching, and mobile health with SMS text reminders [19]. The program content is nonprescriptive; it provided general health messages and focused on small achievable changes around physical activity and eating to enhance self-efficacy and sustainability of behaviour change [22]. There was no reference to knee pain in the intervention. Briefly, the program consisted of (i) week 1: one facilitator-led 60-min interactive group session held with 8–15 women at community locations. The group session aimed to generate a shared understanding and knowledge of health, healthy eating behaviours and physical activity behaviours. Simple messages regarding eating and physical activity behaviours provided an achievable context in which to begin to formulate their own priorities, rather than when or how to perform these behaviours. Facilitators using an interactive model and supported by the program manual, worked through examples of behavioural self-management skills including setting health priorities, problem solving, and self-monitoring, focusing on small changes to behaviour. (ii) Week 2–4: women continue with program manual at home at own pace. The interactive program manual contained assessments, health information, personal stories, activities to challenge personal beliefs and behaviours and opportunities to develop self-management skills such as problem solving and action planning, and tools to self-monitor and assess progress that aimed to develop and improve skills in self-management. (iii) Weeks 2–52: monthly SMS text messages consistent with program messages to remind participants of key behaviours. (iv) Week 12: one 20-min phone coaching session based on motivation interviewing delivered by trained coaches and aimed to assist completion of manual activities and inforce intervention messages and generate action plans [19, 22]. All program activities focused on increasing awareness through personal stories, identifying personal barriers and enablers and developing goals through activities and self-assessments designed to enhance intrinsic motivation and increase self-confidence [22]. Facilitators delivered the intervention including phone coaching after they had completed program-specific training. They were required to have a tertiary qualification in health sciences and undergo 1-day training [22]. The control group received one general women’s health education session based on national healthy diet and activity recommendations, held with 8–15 women at community locations [19, 22].

### Anthropometric data

At baseline and 1-year, weight was measured using calibrated digital scales with participants in light clothing, with an empty bladder, and without shoes, and height was measured using a portable stadiometer. Body mass index (BMI, weight/height^2^, kg/m^2^) was calculated.

### Knee pain

At baseline and 1-year follow-up, knee pain was assessed with the Western Ontario and McMaster Universities Osteoarthritis Index (WOMAC) [23], using a Likert scale (0–4) for each of the five knee pain questions with the WOMAC knee pain score ranging from 0 to 20. Knee pain worsening was defined as any increase in WOMAC pain score from baseline to follow-up. In women without knee pain at baseline (WOMAC pain score = 0), incidence of knee pain was defined if they developed any knee pain at follow-up (WOMAC pain score ≥ 1). In those with any knee pain at baseline (WOMAC pain score ≥ 1), knee pain increasing was defined if their follow-up knee pain score was greater than the baseline measure, while knee pain improvement was defined if their follow-up knee pain score was less than the baseline measure.

### Statistical analysis

With 390 women who provided knee pain data at both baseline and 1-year follow-up, this study had 80% power to detect an absolute risk reduction of 10% in worsening of knee pain between intervention and control groups (10% vs. 20%) with 5% significance level. Baseline characteristics were compared using independent samples *t* test or Mann-Whitney *U* test for continuous variables where appropriate, and chi-squared test for categorical variables between the intervention and control groups. The effect of intervention on knee pain change outcomes (all dichotomous variables) was examined using chi-squared test and binary logistic regression adjusted for age, BMI, town cluster, and baseline knee pain. The interaction of intervention and the presence of baseline knee pain, and interaction of intervention and the baseline overweight status, for their association with knee pain worsening were examined. Subgroup analysis of women who were overweight or obese (BMI ≥ 25 kg/m^2^) at baseline was performed. The number needed to treat, i.e. the estimated number of people who need to be treated in order for one additional person to benefit, was calculated. *P* values of less than 0.05 (two-tailed) were considered statistically significant. All statistical analyses were performed using Stata (Intercooled Stata 12, StataCorp LP., College Station, TX, USA).

## Results

**Fig. 1:**
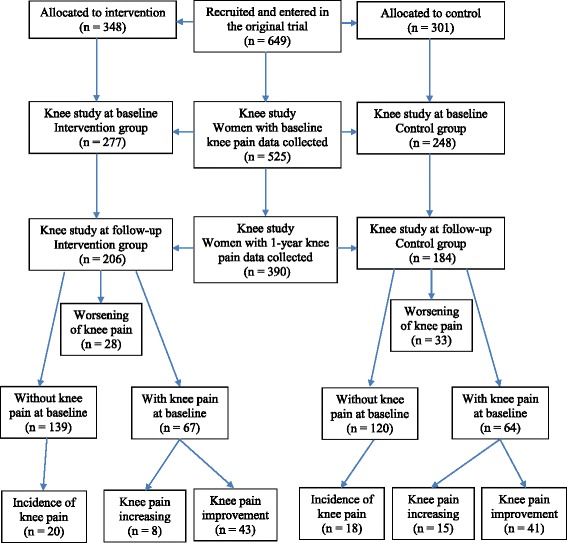
Flowchart of the knee pain substudy

The flowchart for the knee pain substudy is presented in Fig. 1. A total of 649 women were recruited for the original study, of whom 525 (81%) women had complete data for age, BMI, and WOMAC and were included in the current knee pain substudy. There were no significant differences in baseline characteristics between the intervention and control groups (Table 1). There were no significant differences in baseline characteristics between those who were included in the current study and those who were not, except for a difference in BMI (Additional file 1: Table S1). There were 390 (74%) women who had knee pain data collected at 1 year. Of these 390, there were no significant differences in baseline characteristics between the intervention and control groups except for that women in the intervention group were older (*p* = 0.04) (Table 1). Those who did not provide 1-year knee pain data were younger and had greater BMI and lower education level compared with those who provided the data, with no differences observed for employment status and baseline knee pain between the two groups (Additional file 2: Table S2).

**Table 1:** Characteristics of study participants

Baseline characteristics	Intervention^a^ (*n* = 277)	Control^a^ (*n* = 248)	*P* value^c^	Intervention^b^ (*n* = 206)	Control^b^ (*n* = 184)	*P* value^c^
Age, years	40.0 (6.1)	39.0 (7.1)	0.11	40.7 (5.7)	39.4 (6.8)	0.04
Body mass index, kg/m^2^	28.5 (6.1)	28.1 (6.7)	0.50	28.4 (6.3)	27.2 (5.8)	0.06
Employment, n (%)			0.64			0.49
Full-time paid work	52 (19.0)	41 (16.7)		38 (18.5)	27 (14.8)	
Part time/casual work	144 (52.6)	139 (56.5)		110 (53.7)	108 (59.0)	
No paid work	78 (28.5)	66 (26.8)		57 (27.8)	48 (26.2)	
Education, n (%)			0.20			0.05
No post school qualification	42 (15.3)	50 (20.3)		29 (14.2)	38 (20.8)	
Certificate/diploma/apprenticeship	127 (46.2)	116 (47.2)		80 (39.2)	81 (44.3)	
Bachelor degree or higher	106 (38.6)	80 (32.5)		95 (46.6)	64 (35.0)	
WOMAC pain, median (range)	0 (0–20)	0 (0–15)	0.77	0 (0–20)	0 (0–15)	0.76
Any self-reported knee pain^d^, n (%)	98 (35.4)	85 (34.3)	0.79	67 (32.5)	64 (34.8)	0.64

Data presented as mean (standard deviation), median (range), or n (%)

^a^Women with complete data at baseline for age, body mass index, town cluster, and WOMAC

^b^Women with complete data for age, body mass index, town cluster, and WOMAC at baseline, and WOMAC at 1 year

^c^Differences between intervention and control groups using independent samples t-test, chi squared test, or Mann-Whitney U test where appropriate

^d^Defined as baseline WOMAC pain score (range 0–20) ≥ 1

The effect of HeLP-her on knee pain change over 1 year is presented in Table 2. Worsening of knee pain was observed in 28 (13.6%) women in the intervention group and 33 (17.9%) women in the control group (*p* = 0.24). After adjusted for age, BMI, town cluster, and baseline knee pain, the risk of worsening knee pain did not differ between the intervention and control groups (odds ratio [OR] 0.67, 95% CI 0.38–1.17, *p* = 0.16). There was evidence for a potential interaction between intervention and the presence of baseline knee pain for the association with worsening of knee pain (*p* = 0.12). For women with no knee pain at baseline, incident knee pain was observed in 20 (14.4%) women in the intervention group and 18 (15.0%) women in the control group (*p* = 0.89); the risk of incident knee pain did not differ between the two groups after adjusted for age, BMI, and town cluster (OR 0.92, 95% CI 0.45–1.84, *p* = 0.81). For women with knee pain at baseline, knee pain increasing was observed in 8 (11.9%) women in the intervention group and 15 (23.4%) women in the control group (*p* = 0.08), while knee pain improvement was observed in 43 (64.4%) women in the intervention group and 41 (64.1%) women in the control group (*p* = 0.99). After adjusted for age, BMI, town cluster, and baseline knee pain, women in the intervention group had a lower risk of knee pain increasing compared with those in the control group (OR 0.37, 95% CI 0.14–1.01, *p* = 0.05; number needed to treat nine), while the likelihood of knee pain improvement did not differ between the two groups (OR 1.13, 95% CI 0.53–2.43, *p* = 0.75). When the data for the intervention and control arms were pooled and analysed, 38.7% of women had weight gain and 59.9% had weight loss. Weight gain was non-significantly associated with an increased risk of knee pain increasing (OR 1.15 for every 1 kg weight gain, 95% CI 0.99–1.34, *p* = 0.07), and weight loss was non-significantly associated with a reduced risk of knee pain increasing (OR 0.87 for every 1 kg weight loss, 95% CI 0.75–1.01, *p* = 0.07), adjusted for baseline age, height, weight, knee pain, and town cluster.

**Table 2:** Effect of intervention on change in knee pain over 1 year

	Intervention, n (%)	Control, n (%)	*P* value^a^	Odds ratio (95% CI)^b^	*P* value^b^
Whole population, *n* = 390	*n* = 206	*n* = 184			
Knee pain worsening	28 (13.6)	33 (17.9)	0.24	0.67 (0.38, 1.17)	0.16
Subgroup with no knee pain at baseline, *n* = 259	*n* = 139	*n* = 120			
Incidence of knee pain	20 (14.4)	18 (15.0)	0.89	0.92 (0.45, 1.84)	0.81
Subgroup with knee pain at baseline, *n* = 131	*n* = 67	*n* = 64			
Knee pain increasing	8 (11.9)	15 (23.4)	0.08	0.37 (0.14, 1.01)	0.05
Knee pain improvement	43 (64.2)	41 (64.1)	0.99	1.13 (0.53, 2.43)	0.75

^a^Differences between intervention and control groups using chi squared test

^b^Logistic regression, intervention vs control group, adjusted for age, body mass index, town cluster, and baseline WOMAC pain score

There was evidence for a potential interaction between intervention and the overweight/obese status for the association with worsening of knee pain (*p* = 0.03). The effect of HeLP-her on knee pain change over 1 year was further examined in the subgroup of women who were overweight or obese at baseline (*n* = 242) (Table 3). After adjustment for age, BMI, town cluster, and baseline knee pain, women in the intervention arm had a significantly lower risk of knee pain worsening (OR 0.45, 95% CI 0.23–0.87, *p* = 0.02; number needed to treat eight) and knee pain increasing (OR 0.28, 95% CI 0.09–0.87, *p* = 0.03; number needed to treat five) compared with those in the control arm, while the incidence (OR 0.68, 95% CI 0.29–1.62, *p* = 0.39) or improvement (OR 2.16, 95% CI 0.87–5.32, *p* = 0.10) of knee pain did not differ between the two groups. We found similar results for women who were overweight and those who were obese, but the results were less significant due to reduced sample size. For example of the effect of intervention on knee pain worsening with the same adjustment, the OR was 0.45 (95% CI 0.17–1.16, *p* = 0.10) for overweight women and 0.46 (95% CI 0.18–1.20, *p* = 0.11) for obese women.

**Table 3:** Effect of intervention on change in knee pain over 1 year in women with baseline BMI ≥ 25 kg/m^2^

	Intervention, n (%)	Control, n (%)	*P* value^a^	Odds ratio (95% CI)^b^	*P* value^b^
Women who were overweight or obese, n = 242	*n* = 137	*n* = 105			
Knee pain worsening	19 (13.9)	27 (25.7)	0.02	0.45 (0.23, 0.87)	0.02
Subgroup with no knee pain at baseline, *n* = 147	*n* = 85	*n* = 62			
Incidence of knee pain	13 (15.3)	13 (21.0)	0.37	0.68 (0.29, 1.62)	0.39
Subgroup with knee pain at baseline, *n* = 95	*n* = 52	*n* = 43			
Knee pain increasing	6 (11.5)	14 (32.6)	0.01	0.28 (0.09, 0.87)	0.03
Knee pain improvement	36 (69.2)	23 (53.5)	0.12	2.16 (0.87, 5.32)	0.10

^a^Differences between intervention and control groups using chi squared test

^b^Logistic regression, intervention vs control group, adjusted for age, body mass index, town cluster, and baseline WOMAC pain score

## Discussion

In community-based young to middle-aged rural women who were selected without reference to knee symptoms, a simple low-intensity lifestyle intervention (HeLP-her) reduced the risk of knee pain getting worse in those who had any knee pain, particularly in those who were overweight or obese, although no effect of the HeLP-her lifestyle intervention was demonstrated on the overall knee pain change for the whole study sample.

We found the HeLP-her lifestyle intervention reduced the risk of knee pain increasing in women who had any knee pain regardless of their BMI, with a stronger effect shown in overweight or obese women. This equated to nine women with any knee pain needing to engage in the HeLP-her program to prevent one additional woman having an increase in knee pain, and only five overweight/obese women with any knee pain need to engage in the HeLP-her program to prevent one additional woman having an increase in knee pain. The HeLP-her has been established with the aim to prevent weight gain, and tested in community-based young to middle-aged urban (community and antenatal populations and settings) and in rural women [19–21]. The HeLP-her program prevented weight gain with a mild effect on weight loss over 1 year where women in the control group gained weight, resulting in a between-group difference in weight change of approximately 1 kg at 12 months [19, 20]. Obesity and weight gain are established risk factors for knee pain [3, 5, 6]. However the evidence for weight loss and reduced knee pain is limited and largely in populations with established osteoarthritis [12, 13, 18] or co-morbidities such as diabetes [24]. Two randomised controlled trials of overweight or obese participants with osteoarthritis and knee pain demonstrated modest improvement in knee pain after very intensive weight loss programs resulting in 10% or approximately10 kg weight loss over 12–18 months [12, 13]. In overweight or obese adults with diabetes, an intensive lifestyle intervention which resulted in a reduction of 3.1 kg/m^2^ in BMI on average over 12 months, reduced the risk of developing knee pain by 15% at 1 year compared with diabetes support and education, which led to an average of 0.2 kg/m^2^ reduction in BMI which attenuated at 4 years [24]. Such significant weight loss requires intensive, prolonged contact, supervised exercise and dietary modification delivered by a multidisciplinary team [12, 15]. As a result, the adherence to intervention activities is often poor (approximately 50% for adherence to exercise and approximately 60% for adherence to diet intervention over 18 months) and long-term outcomes are disappointing [12, 15, 24]. In contrast, the simple low-intensity HeLP-her program targeted community-based women with the aim of preventing weight gain. While successfully preventing weight gain over 1 year, it was highly acceptable to participants and achievable with low participation burden and good retention at approximately 80% [19, 25]. By performing a substudy with secondary analysis on the original randomised controlled trial [19] with knee pain data prospectively collected using a validated tool, we found a favourable effect of the HeLP-her program on reducing the risk of knee pain worsening over 1 year in community-based young to middle-aged women selected without reference to knee pain, supporting its potential for knee pain prevention.

The study has limitations. Women who provided baseline knee pain data and were included in the current knee pain study had lower BMI than those who were not included; women who completed the 1-year follow-up of current knee study were older, higher educated, and had lower BMI than those who did not complete follow-up. The potential selection bias resulted in those of lower BMI tending to take part in this study so may have underestimated the effect of the interventions on knee pain. Data were not collected regarding work absence or other metrics to illustrate the effect of the Help-her program on function and quality of life. The intervention was over 12 months. While the HeLP-her program showed a beneficial effect on reducing the risk of knee pain worsening at 1 year, its longer term effect will need to be determined. The loss to follow-up of the current knee pain study was 26%, which was within the anticipated range and less than other lifestyle interventions [26]. The strengths of the trial include a community-based rural population which was selected without reference to the presence of knee symptoms, the pragmatic design with few exclusion criteria and high external validity, the large scale of the trial enabling valid subgroup analysis which identified a subgroup of women mostly likely to benefit from the HeLP-her program.

The HeLP-her program targets multiple behaviours with the aim to prevent weight gain through its focus on achievable changes in healthy eating and physical activity behaviours. No component of the program targeted joint health or pain management. The effect of HeLP-her program on reducing knee pain is most likely via its beneficial effect on weight. There is no clear evidence suggesting an effect of leisure time physical activity on knee pain [27, 28]. Although the HeLP-her program showed a modest non-significant effect on leisure time activity and sitting time [19], any effect of the HeLP-her program on reducing knee pain by preventing inactivity is likely to be very small.

Knee pain is a common musculoskeletal complaint, particularly in women [2] with no effective preventive treatments. Obesity is a major public health problem with its rate increasing globally and evidence of a link between excess weight and knee pain [18]. Given adults progressively gain weight, increasing the risk of knee pain and osteoarthritis and that knee pain episode predicts future knee pain [6, 10], early interventions preventing weight gain and knee pain in the community are very important in order to reduce the burden of knee pain and disability. There is an unmet need for a simple intervention that targets prevention of both weight gain and knee pain in the community settings. Rural-dwelling women are disadvantaged with higher rates of weight gain and joint pain [29, 30]. The social inequality is reflected by their poorer health outcomes and limited access to health care. In our study of community-based young to middle-aged rural women, 35% reported any knee pain. The HeLP-her program is a simple low-intensity lifestyle intervention that is feasible to deliver, highly acceptable to participants, and requires few resources and health practitioner time, thus it is highly feasible to be implemented in the community settings. In a series of randomised controlled trials HeLP-her has been shown to prevent weight gain in young to middle-aged women [19–21], with extension out to 2 years and a health economic analysis of HeLP-her rural still underway. Our study showed a favourable effect of this lifestyle program in the HeLP-her rural study on reducing the risk of knee pain worsening in community-based young to middle-aged women who were selected without reference to knee pain or disease. There is an urgent and unmet need for a new approach aimed at preventing knee pain since knee pain is a fluctuating condition and episodes of knee pain predict future knee pain both in terms of number of episodes and increasing severity [10]. Knee osteoarthritis is a major cause of pain, disability and healthcare costs, with no treatments that slow the disease progression. Knee pain is part of a disease cycle such that knee pain is associated with weight gain and quadriceps muscle weakness [10, 16], each in turn risk factors for knee pain [6, 17], structural knee damage, onset and progression of knee osteoarthritis [11], and so further pain and disability. Furthermore, once knee osteoarthritis is established, even major weight loss of over 10% did not slow the structural disease progression [14]. Thus intervening at an early stage of this cycle is important and likely to have a major effect on reducing long-term pain and disability as knee pain and each of the above consequences are associated with the development and progression of knee osteoarthritis [11]. It is well-established that increased weight is a risk factor for knee osteoarthritis [11] and that over midlife women tend to steadily gain weight [7]. In this study we showed that the HeLP-her had an effect on preventing knee pain in community-based women with low numbers needed to treat (from five to nine) in order to prevent one woman from having an increase in her knee pain, as described above. As there have been no previous lifestyle intervention programs that are effective in reducing knee pain in community-based population of individuals, the HeLP-her program integrated with a whole population approach may provide a novel, simple feasible strategy of early intervention for reducing the burden of knee pain in the community, especially where resources are limited in rural communities.

## Conclusions

Our results showed that a simple low-intensity lifestyle program was able to reduce the risk of knee pain worsening in a general population of young to middle-aged rural women who had any knee pain, particularly in those overweight or obese. While the favourable effect of the program will need to be confirmed in other populations and to be tested over a longer time period, pragmatic lifestyle programs such as HeLP-her may represent a novel, feasible lifestyle intervention to reduce the burden of knee pain in the community.

## Additional files

Additional file 1: Characteristics of study participants, according to whether or not they had baseline knee pain data. (DOCX 18 kb)

Additional file 2: Characteristics of study participants with baseline knee data, according to whether or not they had knee pain data at 1 year follow-up. (DOCX 18 kb)

## Abbreviations

BMI: Body mass index
CI: Confidence interval
CONSORT: Consolidated Standards of Reporting Trials
HeLP-her: Healthy Lifestyle Program for women
OR: Odds ratio
WOMAC: Western Ontario and McMaster Universities Osteoarthritis Index
