# Supplementary material for: Effect of a low-intensity, self-management lifestyle intervention on knee pain in community-based young to middle-aged rural women: a cluster randomised controlled trial
Source: Arthritis Res Ther. 2018 Apr 17;20:74. doi: 10.1186/s13075-018-1572-5 (PMC5905125; doi:10.1186/s13075-018-1572-5)
Supplement: Supplementary file 1 — Characteristics of study participants, according to whether or not they had baseline knee pain data. (DOCX 18 kb) [file 13075_2018_1572_MOESM1_ESM.docx]

**Table S1: Characteristics of study participants, according to whether or not they had baseline knee pain data**

| **Baseline characteristics** | **With baseline knee pain data**  **n = 525** | **Without baseline knee pain data**  **n = 113** | **P value^a^** |
| --- | --- | --- | --- |
| Age (years) | 39.5 (6.6) | 39.9 (7.9) | 0.69 |
| Body mass index (kg/m^2^) | 28.3 (6.4) | 30.3 (7.5) | 0.004 |
| Employment, n (%) |  |  | 0.54 |
| Full time paid work | 93 (17.9) | 13 (23.6) |  |
| Part time/casual work | 283 (54.4) | 29 (52.7) |  |
| No paid work | 144 (27.7) | 13 (23.6) |  |
| Education, n (%) |  |  | 0.12 |
| No post school qualification | 92 (17.7) | 13 (24.1) |  |
| Certificate/diploma/apprenticeship | 243 (46.6) | 29 (53.7) |  |
| Bachelor degree or higher | 186 (35.7) | 12 (22.2) |  |

Data presented as mean (standard deviation) or n (%)

^a^differences between intervention and control groups using independent samples t-test or chi squared test
