# Supplementary material for: Effect of a low-intensity, self-management lifestyle intervention on knee pain in community-based young to middle-aged rural women: a cluster randomised controlled trial
Source: Arthritis Res Ther. 2018 Apr 17;20:74. doi: 10.1186/s13075-018-1572-5 (PMC5905125; doi:10.1186/s13075-018-1572-5)
Supplement: Supplementary file 2 — Characteristics of study participants with baseline knee data, according to whether or not they had knee pain data at 1 year follow-up. (DOCX 18 kb) [file 13075_2018_1572_MOESM2_ESM.docx]

**Table S2: Characteristics of study participants with baseline knee data, according to whether or not they had knee pain data at 1 year follow-up**

| **Baseline characteristics** | **Completer**  **n = 390** | **Non-completer**  **n = 135** | **P value^a^** |
| --- | --- | --- | --- |
| Age (years) | 40.1 (6.3) | 37.8 (7.2) | 0.0004 |
| Body mass index (kg/m^2^) | 27.8 (6.1) | 29.7 (7.2) | 0.003 |
| Employment, n (%) |  |  | 0.34 |
| Full time paid work | 65 (16.7) | 28 (21.2) |  |
| Part time/casual work | 218 (56.2) | 65 (49.2) |  |
| No paid work | 105 (27.1) | 39 (29.6) |  |
| Education, n (%) |  |  | <0.001 |
| No post school qualification | 67 (17.3) | 25 (18.7) |  |
| Certificate/diploma/apprenticeship | 161 (41.6) | 82 (61.2) |  |
| Bachelor degree or higher | 159 (41.1) | 27 (20.1) |  |
| WOMAC pain, median (range) | 0 (0-20) | 0 (0-11) | 0.35 |
| Knee pain, n (%) | 131 (33.6) | 52 (38.5) | 0.30 |

Data presented as mean (standard deviation), median (range), or n (%)

^a^differences between intervention and control groups using independent samples t-test, chi squared test, or Mann-Whitney U test
